# Cell culture media dependent in vitro dynamics and culture characteristics of adult caprine dermal fibroblast cells

**DOI:** 10.1038/s41598-023-38634-4

**Published:** 2023-08-22

**Authors:** Juhi Pathak, Shiva Pratap Singh, Suresh Dinkar Kharche, Anjana Goel, Yogesh K. Soni, Rakesh Kaushik, Megha Kose, Ashish Kumar

**Affiliations:** 1 Animal Physiology and Reproduction Division, ICAR-Central Institute for Research on Goats, Makhdoom, Farah, Mathura, Uttar Pradesh 281 122 India; 2 Department of Biotechnology, GLA University, Mathura, Uttar Pradesh 281 406 India; 3 Animal Genetics and Breeding Division, ICAR-Central Institute for Research on Goats, Makhdoom, Farah, Mathura, Uttar Pradesh 281 122 India

**Keywords:** Biological techniques, Cell biology

## Abstract

The enhanced availability of functional fibroblasts from precious tissue samples requires an ideal cell-culture system. Therefore, this study was designed to investigate the performance of caprine adult fibroblast cells (cadFibroblast) when cultivated in different culture media. The cadFibroblast cell lines from adult Barbari (*Capra hircus*) bucks were established and the effect of different media viz*.* DMEM/F-12 [with low-glucose (5.5 mM; DL) and high-glucose (30 mM; DH)], *α*-MEM [with low-glucose (5.5 mM; ML) and with high-glucose (30 mM; MH)], and fibroblast growth medium (FGM) were evaluated. Cells were then compared for growth characteristics and in-vitro dynamics through cellular morphology, proliferation, population-doubling time, double-immunocytochemistry, colony-forming units, wound healing, transwell migration, and differential expression of fibroblast-specific markers (FSP-1 and vimentin). The results of immunocytochemistry, transwell migration/invasion, and wound healing assays showed the superiority of DH over DL and other media tested. Whereas, similar effects of glucose supplementation and expression of FSP-1 were not observed in *α*-MEM. Transwell migration was significantly (*p* < 0.05) lower in FGM compared with other media tested. Overall, our results illustrate the media-dependent deviation in in-vitro dynamics and culture characteristics of cadFibroblasts that may be useful to develop strategies to cultivate these cells efficiently for research and downstream applications.

## Introduction

Fibroblasts, the most prevalent cells of the dermis, are associated with maintaining structural homeostasis^1^ as well as the synthesis of growth factors, and extracellular matrix proteins such as collagen, elastin, and fibronectin^2^. The establishment of fibroblast cell lines and their application in understanding cellular protein or gene functions makes a significant contribution to the efficient preservation and use of genetic resources. The isolation and cultivation of fibroblasts from different animal tissues for the establishment of fibroblast cell lines are commonly used methods for the preservation of live-tissue genetic materials^3^. Thus, the successful establishment of fibroblast cell lines and their cryopreservation may serve as a complementary strategy for the conservation of genetic resources of valued and endangered animals. The fibroblasts play a crucial role in the process of usual development, tissue maintenance, wound repair, angiogenesis, and several other biological processes such as cell migration, differentiation, and apoptosis by producing several regulatory molecules including cytokines and growth factors, and crosstalk with the other cell populations. These cells also hold considerable promise for developing animals that have undergone genetic modification^4,5^. Due to their usage as nuclear donors in the SCNT procedure, fibroblasts are also the ideal choice for preserving endangered species and animal germplasm with superior genetics.

These cells from many species have been successfully cultured *in vitro*^6–9^. The establishment of fibroblast cell lines that are particular to a species, tissue, or disease and have a high rate of proliferation, lifespan, and genetic stability is crucial for the success of such studies^9^. Developing such fibroblast culture systems will also enable the researchers to get representative cells that have retained most of their original features and functions, providing a crucial basic framework for additional cell engineering and cell biology^10^.

The recent advancement of gene delivery methods for the production of genome-edited animals resulted in increased demand for species-specific genetically modified cell lines. Furthermore, despite sharing comparable features, tissue-specific fibroblast lineages exist to support the improved developmental, homeostatic, and repair needs of specific organs. However, because of their short lifespan, fibroblasts are also a limited resource for their applications. Thus, suitable culture media is required to ensure their ideal growth performance during primary culture and throughout subsequent passages as most mammalian cells in in vitro culture systems require specialized media supplemented with a complex of nutrients and growth factors.

The available literature fails to demonstrate the effect of culture media on caprine adult dermal fibroblast cells (cadFibroblast) through biological processes like proliferation, formation of colony forming unit (CFU), and cell migration. This information is important as cell adhesion, proliferation, and migration are the basis for several pathophysiological processes involving tissue remodeling, wound healing, chemotaxis, etc. Moreover, research focused to identify the effect of glucose, one of the most vital molecules in biochemistry, on culture characteristics of fibroblast cell culture is unavailable. Most of the classical cell culture media developed so far are for small-scale low-density cultures. Whereas, for clinical applications in the field of regenerative medicine such as the treatment of various non-healing wounds and disease modeling after cell reprogramming, higher cell growth rates with suitable viability of cells are important. Therefore, for research and clinical applications of dermal fibroblast cells, it is essential to optimize culture conditions and media that can support enhanced cell growth and sustain cell viability. The presence of glucose in a cell culture medium may be important and more useful for overall cellular health than it appears.

Therefore, the present study was designed to identify the most appropriate media condition and the added effects of glucose on the proliferation and maintenance of functional properties of cadFibroblast cells. For this, we first carefully isolated and cultured the cadFibroblast cells in different culture systems, and then the pattern of cell proliferation, population doubling time (PDT), fibroblast marker expression [fibroblast specific protein-1 (FSP-1), and vimentin; through double immunocytochemistry and differential gene expression], formation of colony forming unit (CFU), scratch wound healing, and trans-well migration were evaluated and compared. The commercially available cell culture media (Dulbecco’s Modified Eagle’s Medium/Nutrient Mixture F-12 Ham (DMEM/F-12; with low and high glucose supplementation), Minimum Essential Medium Alpha (*α*-MEM; with low and high glucose supplementation), and Fibroblast Growth Medium (FGM) were compared.

In this study, we for the first time demonstrate the results of the media-dependent effects of glucose supplementation and the expression of FSP-1 in the cultured fibroblasts through immunocytochemical localization and gene expression analyses. The outcome of the study may further be utilized for the characterization and enrichment of the skin fibroblast cells of other animal species and their downstream applications in areas of regenerative medicine such as treatment of various non-healing wounds, disease modeling after cell reprogramming, and production of genome-edited animals.

## Results

### Primary cell cultures

**Figure 1:**
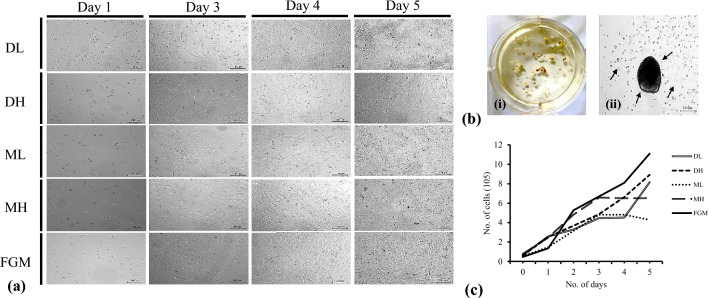
Effect of culture media on proliferation of caprine adult dermal fibroblast cells (cadFibroblast). (**a**) Comparison of microscopic aspects of cadFibroblast in different culture media from the day (d) 1 to d 5 in culture. Cells in all culture media displayed spindle-like typical fibroblast morphology except for FGM with more elongated cells and sharper needle-like morphology. (**b**) Caprine tissue explant culture and outgrowth of adult dermal fibroblasts from explants (P-0). [**b**(**i**)] Caprine skin explants after mincing in culture media were transferred in 6 well cell culture plates and incubated at 38.5 °C, 5% CO_2_ in humidified conditions for tissue adherence and attachment [**b**(**ii**)] Illustrates a caprine skin explant with the migration of spindle-shaped fibroblast cells (black arrows) at d 8 of culture. Dark-shaded areas in the image are the skin explants. (**c**) Proliferative potential of cells was determined by counting the cell number at d 1 to d 5 by automatic cell counter. DL = DMEM with low glucose (5.5 mM), DH = DMEM with high glucose (30.0 mM), ML = *α*-MEM with low glucose (5.5 mM), MH = *α*-MEM with high glucose (30.0 mM), and FGM = Fibroblast growth medium. Scale bar = 100 µm.

The growth of cadFibroblasts was observed only in the vicinity of the attached explants. In this investigation, the development of cadFibroblast cells never occurred in any of the floating explants (Fig. 1b(i)). The cells were spreading around the different explants in different densities (Fig. 1b(ii)]. It was more prevalent in certain explants in comparison to others. We also observed that after the 5th day, on replacing the culture media, 15–20% of the skin explants floated or became detached.

On days 8–10, when these cells reached a confluence of around 60–70% around the explants, the first subculture of the primary culture was performed. The secondary cultures grew considerably more quickly than primary cultures for 70–80% confluence, which may be related to their adaptability to the cultural environment. The secondary culture reached 70–80% confluence much earlier (in 4–5 days) compared with the primary culture, which needed 8–10 days from the day of seeding. The results of culture media-dependent growth pattern of fibroblasts during d 1–5 in culture is presented in Fig. 1a, b.

### Morphology of cadFibroblast cells

In all media, cells expanded normally and formed a monolayer during different days of culture. The cadFibroblast displayed a classic fusiform morphology with oval nuclei positioned in the center, radiating fibroblast-like structures, and migratory patterns resembling flames. Cell morphology was indistinguishable in all the media tested except for FGM with more elongated cells and sharp needle-like morphology.

### Microorganism contamination detection

Every 2nd day of culture, we examined if there was any microbial contamination in cultures, as microbial contamination could lead to cell culture failure. Unsurprisingly, neither branched mycelium of fungi nor any actively moving bacteria were identified on observing under the microscope. Furthermore, no abrupt change in the turbidity of culture media was observed indicating no microbial multiplication during the cultivation of cells.

### Cell proliferation assay and population doubling time

The number of viable cadFibroblast cells in DL, DH, ML, MH, and FGM during different days in culture are presented in Table 1. This shows that optimal culture conditions were used to proliferate the cells and all the media were appropriate (Fig. 1a). Further, we observed that during the initial days of culture (d 0–2), the cells in FGM had a lower proliferation rate in comparison to other media but in later days (d 3–5) they grow exponentially.

**Table 1:** The proliferation of cadFibroblast cells grown in different media during different days in culture.

Time	Number of cells per well (× 10^5^)				
	DL	DH	ML	MH	FGM
Day 0	0.63	0.60	0.55	0.77	0.46
Day 1	2.58	2.52	1.47	2.46	1.35
Day 2	3.29	3.68	3.15	4.82	5.27
Day 3	4.46	4.86	4.81	6.57	6.69
Day 4	4.52	6.63	4.81	6.51	8.09
Day 5	8.15	8.92	4.28	6.52	11.10

The number of cells per well (× 10^5^) was counted in each well of the 24-well cell culture plate.

During the initial phase of growth (d 0–1), lower PDT in DL (11.80) and DH (11.59) was observed compared with ML and MH (16.92 and 14.32, respectively) stating higher proliferation of cells in DL and DH media within 24 h of seeding. During this phase (lag phase), FGM, despite being a readymade media, showed higher PDT (15.45) in comparison to DL and DH. Furthermore, in the log phase (d 1–3), media differed comparatively for the PDT with the lowest PDT in FGM (20.79). PDT in DL (60.78), and DH (45.69) was higher than ML (28.07) and MH (33.87). Similar differences remained continued between d 3–5, where DL (55.19) and DH (54.79) showed the lowest PDT among the groups, affirming higher multiplication of cells in these media. FGM also showed a comparatively higher PDT (65.71) while ML and MH showed a decrease in cell number at d 5 which could be due to the contact inhibition. Overall, cell proliferation in DH was comparatively higher than in DL. These results indicate the superiority of DMEM/F-12 over other media on cell proliferation of cadFibroblast cells.

When the total number of cadFibroblast cells (at different periods) was plotted against time (days), an ‘S-shaped’ growth curve was seen with all of the tested media. After seeding, there was a lag period that can be observed in the graph, which corresponds to the curve's recovery and adaption phase. In the post-recovery period, cells multiplied quickly initiating the log or exponential phase. However, the stationary phase was noticed much earlier (at d 3–4) in ML and MH compared with the other culture media (Fig. 1c).

### Immunocytochemical characterization

**Figure 2:**
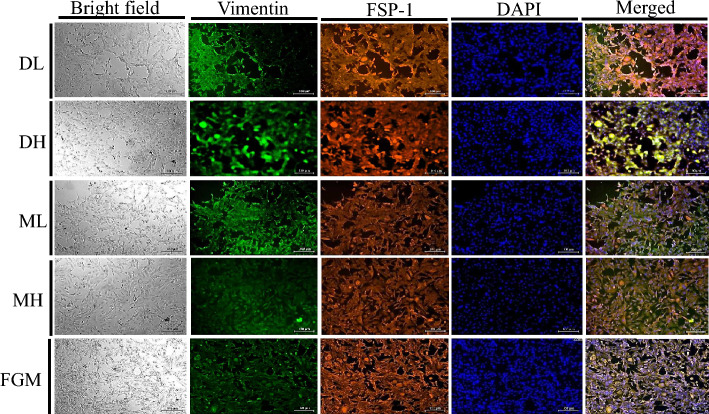
The representative images for characterization of caprine adult dermal fibroblasts by immunostaining. Relative fluorescence positivity with vimentin and Fibroblast Specific Protein (FSP-1) was observed in different culture media. Magnification × 10. DL = DMEM with low glucose (5.5 mM), DH = DMEM with high glucose (30.0 mM), ML = *α*-MEM with low glucose (5.5 mM), MH = *α*-MEM with high glucose (30.0 mM), and FGM = Fibroblast growth medium. Scale bar = 100 µm.

The representative images of double immunofluorescence staining for fibroblast markers i.e. vimentin and FSP-1 are shown in Fig. 2. The immunocytochemical study indicates the higher expression of both the markers (vimentin and FSP-1) in cadFibroblast cells cultured in all the media tested. We observed no difference in the expression of both markers among different media. This implies that the culture media used for the experiment supported the maintenance of normal characteristics and expansion of cadFibroblast during the culture period.

### Colony forming unit (CFU) assay

**Figure 3:**
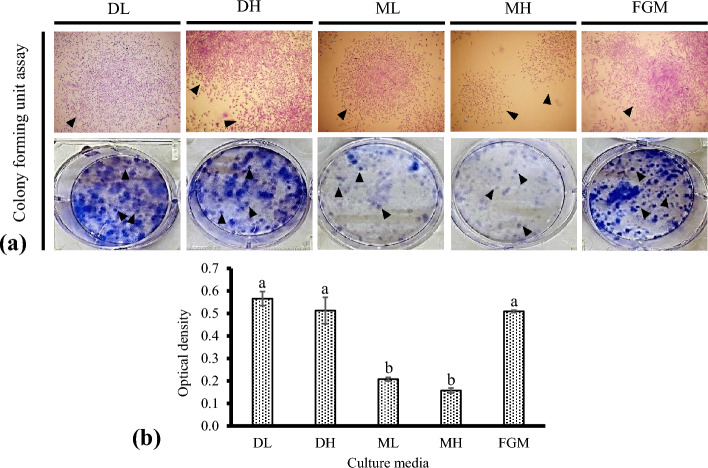
Representative pictures of the effect of culture media on CFU efficiency of caprine adult dermal fibroblast cells (cadFibroblast). (**a**) Approximately 50,000 cells per well in different culture media were cultured in 6-well plates for 48 h in a CO_2_ incubator with 5% CO_2_ at 38.5 °C. Large, round, and compact colonies were observed in all the groups. (**b**) Images showing the CFU ability of cadFibroblasts by 1% crystal violet staining. The optical density in different media for CFU assay was evaluated after solubilization of the crystal violet stain. Arrowheads indicate individual colonies. Bars with dissimilar presentations (a and b) are significantly (*p* < 0.05) different. DL = DMEM with low glucose (5.5 mM), DH = DMEM with high glucose (30.0 mM), ML = *α*-MEM with low glucose (5.5 mM), MH = *α*-MEM with high glucose (30.0 mM), and FGM = Fibroblast growth medium.

In low-density, discrete colonies of adherent cadFibroblasts were formed, every individual colony arising from a single precursor cell, known as CFU. Images of six-well plates containing colonies of cadFibroblast cells in different culture media were acquired (Fig. 3a). Images clearly showed differences in colony formation confirming the effect of media on colony formation.

On comparing the OD (optical density) after solubilizing crystal violet stain in 1% SDS, significantly (*p* < 0.05) higher absorbance was observed in DL (0.57 ± 0.03) and DH (0.51 ± 0.06) in comparison to ML (0.21 ± 0.01) and MH (0.16 ± 0.01) (Fig. 3b) indicating a significantly (*p* < 0.05) higher number of colonies in DL and DH with compared to ML and MH. It could also be stated that an almost similar number of colonies were observed in DL, DH, and FGM media as no significant difference was observed between the ODs of DL (0.57 ± 0.03), DH (0.51 ± 0.06), and FGM (0.51 ± 0.01). These results demonstrate the equal competence of DMEM/F-12 and FGM media for CFU of cadFibroblast cells.

### Wound healing and transwell migration/invasion assay

**Figure 4:**
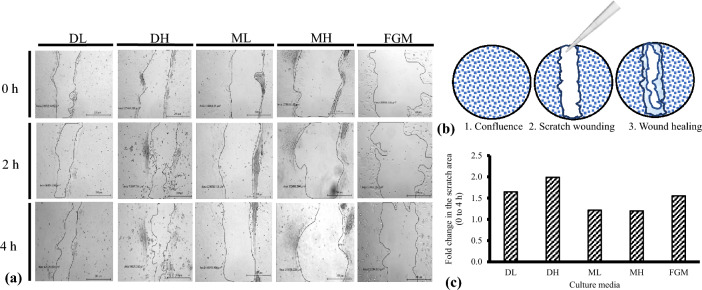
Wound healing assay (scratch assay) using caprine adult dermal fibroblast cells (cadFibroblast). (**a**) Representative pictures of the scratch assay at 0, 2, and 4 h with area measurement (µm^2^). Scale bar = 200 µm. Approximately 50,000 cells/well in different culture media were cultivated in 24-well cell culture plates until nearly confluent to achieve growth arrest. The confluent monolayer of cadFibroblast was then scraped with a sterile small pipette. Cells migrate to cover cell-free areas. To determine the cell migration during scratch closure, the area of each scratch was traced and analyzed using an image-processing algorithm. (**b**) Schematic representation of the wound healing assay. The wound was created by scratching of the confluent cell-covered surface with a standard 200 µl pipette tip. (**c**) Quantification of healing speed area (µm^2^) in different culture media. Results represent fold change (0 to 4 h). DL = DMEM with low glucose (5.5 mM), DH = DMEM with high glucose (30.0 mM), ML = *α*-MEM with low glucose (5.5 mM), MH = *α*-MEM with high glucose (30.0 mM), and FGM = Fibroblast growth medium.

To determine whether the medium could influence cell movement, a wound healing experiment, and transwell migration assay were performed. As wound closure was studied by taking pictures at different time points (Fig. 4a), based on the area of the wound, we estimated the effect of different culture media on the cells migration to close the wound created (Table 2). The schematic representation of wound creation and healing assay is presented in (Fig. 4b). As shown in Fig. 4c, media affects the migration of cells in turn affecting the wound-healing property of cadFibroblast cells. As fold-change is a measurement that indicates how much the final value has changed against the initial point, we calculated the decrease in the area (wound closure) in terms of fold-change. DH showed maximum fold-change (1.99) depicting the highest rate of wound closure and movement of cells in this media. Moreover, DL and FGM showed almost similar results with a fold-change of 1.65 and 1.55, respectively. Whereas, relatively lower values were observed for ML (1.22) and MH (1.20). In all the groups, the monolayer attained 100% confluence after 24 h indicating complete healing of the wounds.

**Table 2:** Results presenting cell migration of cadFibroblasts in different culture media through scratch wound healing assay.

Time interval (h)	Wound area (µm^2^)				
	DL	DH	ML	MH	FGM
0	78,222.4	107,464.3	134,068.1	179,566.2	203,926.3
2	56,454.4	72,097.25	129,358.7	150,080.5	173,433.1
4	47,529.9	54,026.51	110,255.9	149,796.2	131,344.9

The wound area (µm^2^) was measured at different time intervals [immediately upon scratching (0 h), 2 h, 4 h, and 24 h] using digital photographs of selected culture areas.

**Figure 5:**
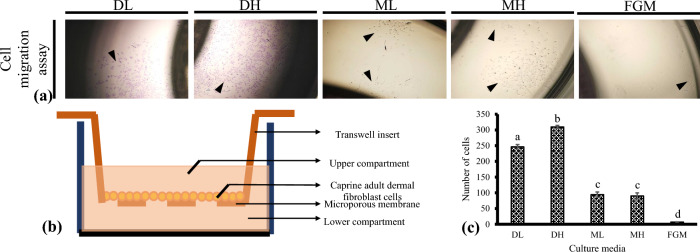
Representative pictures of chemo-attractant-driven cell migration/transwell assay. (**a**) Caprine adult dermal fibroblast cells (cadFibroblast; approximately 1 × 10^6^ cells/well) in different media (100 μl) were placed in the insert’s top chambers and respective media (600 μl) containing 10 ng/ml epidermal growth factor were dispensed to the respective lower chambers of wells as the chemotactic factor. Cells were allowed to migrate for 48 h. After migration, staining of cells was performed with crystal violet, and the number of migrated cells (purple-stained) was counted in ten randomly selected fields using a bright field microscope with a 4 × objective (40× total magnification). Arrows indicate migrated cadFibroblast. (**b**) A diagrammatic representation of the transwell insert device is applied to evaluate cell migration. The small gaps between the lines represent the pores of the membrane. (**c**) Quantitative evaluation of cells moved towards the chemo-attractant (average of 10 random fields at 40 × magnification). Results are expressed as mean ± SEM. Arrowheads indicate migrated cells. Bars with dissimilar presentations (a, b, c, and d) are significantly (*p* < 0.05) different. DL = DMEM with low glucose (5.5 mM), DH = DMEM with high glucose (30.0 mM), ML = *α*-MEM with low glucose (5.5 mM), MH = *α*-MEM with high glucose (30.0 mM), and FGM = Fibroblast growth medium.

To explore the ability of cadFibroblast cells to directional response to chemoattractant, we performed a transwell migration assay. For this, cadFibroblast cells in different media were seeded in the upper chamber and respective media with chemoattractant (EGF) were added at the bottom chambers. Total migrated cells in each group were stained with crystal violet (Fig. 5a, b) and counted in ten randomly selected fields of the fixed cells. In comparison, a significantly (*p* < 0.05) higher number of cells migrated in DH (309.51 ± 0.06) in comparison to DL (246.00 ± 7.47), ML (94.00 ± 8.45), MH (90.00 ± 9.66). Furthermore, cell migration in FGM (6.80 ± 0.97) was significantly (*p* < 0.05) lower when compared with DL, DH, ML, and MH. The slowest migration observed in FGM media could be due to the additional growth factors/supplements present in FGM which decreased the gradient formed.

These results convincingly illustrate that DMEM/F-12 showed higher cell migration of cadFibroblast cells in wound healing and transwell migration assays compared with other media. Furthermore, the high glucose supplementation increased the cell migration in both assays (DH vs DL). However, glucose-induced cell migration could be the base medium dependent as ML and MH did not show any significant difference (Fig. 5c).

### Differential expression of fibroblast-specific marker genes

**Figure 6:**
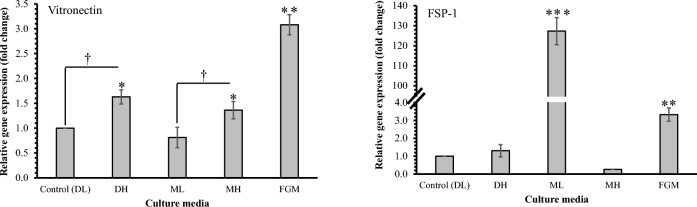
Comparative evaluation of transcription levels of fibroblast-specific marker genes [vimentin and fibroblast specific protein-1 (FSP-1)] in cadFibroblast cultured in different cultured media at P-4 culture. The differential expression levels were assessed using real-time qPCR with GAPDH as an internal calibrator and DL as a control. The significant differences in other groups compared with the control group (DL) are presented by * *p* < 0.05, **p < 0.01, and ***p < 0.001; and † specify glucose-dependent significant (*p* < 0.05) difference between the groups. cadFibroblast = caprine adult dermal fibroblast cells; qPCR = quantitative polymerase chain reaction; DL = DMEM with low glucose (5.5 mM), DH = DMEM with high glucose (30.0 mM), ML = *α*-MEM with low glucose (5.5 mM), MH = α-MEM with high glucose (30.0 mM) and FGM = Fibroblast growth medium.

The product length of the FSP-1 and vimentin gene was validated using 2.0% agarose gel electrophoresis. The amplicon size of FSP-1 and vimentin was 115 and 250 base pairs. Further, the relative quantification of the expression level of FSP-1 and vimentin genes in cadFirboblasts cultivated in different culture media during P-4 was analyzed and compared (Fig. 6).

The expression level of FSP-1 was analyzed in cadFibroblast. The relative expression of the FSP-1 gene in cadFibroblasts grown in DH, ML, MH, and FGM exhibited 1.30 ± 0.34, 127.31 ± 6.76, 0.26 ± 0.00, and 3.32 ± 0.37-fold change as compared to the calibrator (DL). Whereas, in comparison to the control, relative quantification of the vimentin gene in cadFibroblast from DH, ML, MH, and FGM showed 1.63 ± 0.14, 0.81 ± 0.20, 1.36 ± 0.17, and 3.08 ± 0.20-fold changes (Fig. 6).

## Discussion

The present study determines the effect of culture media effect and glucose supplementation on parameters related to proliferation, colony-forming unit, and migration process (wound healing and transwell migration). The key benefits of the in vitro assays performed in this study are that they are simple to use, quick, accurate, and have excellent reproducibility. The migration-related experiments done may also provide insightful details about the possibility of spreading cancer-associated fibroblasts.

We observed that cadFibroblasts from tail tissue can be harvested and subcultured without affecting the health of the donor animal. The first outgrowth from the explants was diverse, according to morphological observations. The cells emerged from the edges of tissue pieces explants around a week post-attachment to the cell culture plates. This was in line with the duration that was described in the literature for the emergence of fibroblasts in different species^11^. We observed that cells with elongated and spindle-like shapes rapidly emigrated from the skin tissue explants. However, although being well attached to the plastic, the cells were sensitive to trypsin treatment. These characteristics demonstrated the fibroblastic origin of the cultured cells^12^.

During the initial culture of skin tissue explants, the main objective was to obtain cadFibroblast cells rather than other potential cell types including epithelial cells. However, fibroblast cells expanded more than epithelial cells, which were barely present. During the detachment of cells by partial trypsinization of three to five minutes, the cadFibroblasts detached from the plastic surface and turned into a spherical shape. Whereas, epidermal cells remained adhered to the bottom and sustained their membranous and polygonal form. After passaging, the cadFibroblast cells expanded progressively after culturing and developed quickly.

It is stated that compared to epithelial cells, fibroblasts attach and trypsinized more quickly^6^. They have a greater development capacity and gradually overtake their epithelial counterparts with serial passaging^13^. These properties enable the development of a pure fibroblast culture after three to four passages. According to previous reports, frequent trypsinization may affect biological properties, particularly those defining genetic traits^13^. Contrary to immortalized cells, fibroblast cells undergo only a certain number of passages before they reach senescence in an in vitro environment^14^. Therefore, in this study, only early passage cells (P4–P6) were used for all experiments.

The partial or fast trypsinization method was used to obtain a purified population of cadFibroblasts^15^. Long-term incubation with high trypsin concentration may cause damage to cells. Therefore, we used a short exposure period (approx. 3 min) with a low concentration of Trypsin EDTA (0.25%) for partial trypsinization and it can be used for frequent passaging without affecting the biological properties of cells. While conducting partial detachment of cells in primary culture through selective trypsinization using 0.25% Trypsin–EDTA solution, cadFibroblasts become spherical and are detached from the surface as single cells in 3–5 min. at 37 °C. Whereas, epithelial cells leave the surface as large aggregates or sheets and require 15 to 30 min to detach^16^. Moreover, fibroblasts can be clearly distinguished from epithelial cells based on their distinct morphological characteristics. The fibroblasts were scattered, elongated, spindle-shaped, and uniform in appearance. Whereas, epithelial cells were having their typical polygonal shape and islets in different sizes^17^. The cultured cells were further enriched by the immunomagnetic separation method (MACS) and characterized through immunocytochemical staining with fibroblast-specific markers (vimentin and FSP-1) to examine the fibroblastic characters of the enriched cells. The purified cadFibroblasts expressed strong fibroblast-specific intracellular (vimentin) and surface (FSP-1) marker expression (Fig. 2).

The total cell count (cell proliferation) in the FGM media (1.35 × 10^5^/ml) was comparatively lower than that observed in the DL (2.58 × 10^5^/ml), DH (2.52 × 10^5^/ml), ML (1.47 × 10^5^/ml), and MH (2.46 × 10^5^/ml) media during the initial phase of the growth (d 1). It was quite a perplexing observation as FGM is a commercially available ready-made media designed for fibroblast culture. The FGM had a smaller growth lag phase, which corresponds to the adaption and recovery time of the cells against trypsin damage.

However, in the log phase (d 1–3), media differed comparatively concerning the PDT with the lowest PDT in FGM (20.79). The PDT in DL (60.78), and DH (45.69) was higher than ML (28.07) and MH (33.87). However, a difference was observed between d 3–5, where DL (55.19) and DH (54.79) showed the lowest PDT among the groups affirming higher multiplication of cells in these media. The FGM also displayed a comparatively higher PDT (65.71) while ML and MH showed a decrease in cell number at d 5 which could be due to the contact inhibition.

Our results are in accordance with the findings of previous studies which describe PDT between 22 and 25 h for various species of highly proliferative fibroblast cells^7,9,13^. The results showed that the growth curve in all the media was similar but not identical. Additionally, the growth curve reveals that the maximum number of cadFibroblast cells could be obtained on day five of the culture, regardless of the media.

Colony formation varies by many contributing factors like laboratory conditions, baseline media, serum, culture handling, counting technique, and the addition of growth factors and supplements^18^. In the present study, we also investigated how culture media could affect overall CFU results. On comparing the OD after solubilizing crystal violet stain with 1% SDS, significantly (*p* < 0.05) higher absorbance was observed in DL and DH compared with ML and MH indicating a significantly higher number of colonies in DL and DH. Further, it could be stated that an almost similar number of colonies were present in DL, DH, and FGM media as no significant difference among the ODs of DL, DH, and FGM was observed.

The process of cell migration is crucial because that allows cells to adapt and move into the correct position or destination in an environment to execute their functions^19^. Cell migration is the directed movement of a single cell or a group of cells in response to chemical and/or mechanical signals. To initiate migration, individual cells receive signals, which set in motion the complex and highly coordinated molecular machinery that drives these cells to move in the right direction with the appropriate speeds and to arrive at their destinations. In multicellular organisms, this process is essential for gastrulation, immune cell trafficking, tissue homeostasis, embryonic morphogenesis, and nervous system development. Moving cells can, however, become out of control and contribute to a variety of pathological events, including cancer and inflammation^20–22^. The in vitro experiments are great tools for studying the behavior of live cells and extrapolating results to in vivo conditions. In vitro cell migration and wound closure tests provide detailed information about the migratory behaviors of cells. Therefore, they can be utilized to investigate the molecular causes of cell migration also^23–25^.

In the domains of physiology and oncology, methods for examining cell migration are interesting since these traits are important for determining how new therapeutic medicines and chemoattractants affect the spread of metastatic disease. The healthy and faster healing process depends on fibroblast migration from the wound edge to the wounded area^26^. Therefore, the cell wound closure assay evaluates the capacity of the cell line to move and subsequently heal a wound created in a confluent cell plate. Additionally, transwell migration examines the capacity of fibroblast cells to attach to the extracellular matrix (ECM) and directionally respond to chemoattractants and treatments.

Here, we also investigated the culture medium-dependent cadFibroblast migration through in vitro wound closure migration experiment. As the rate of cell migration in an in vitro wound model can be measured using this in vitro scratch assay through its easy and uncomplicated procedure^27^. Therefore, we used this assay to examine cadFibroblast migration and observed that the DH medium showed the maximum migration of cells for wound closure after 4 h. Moreover, no difference in the cell migration was detected between DL and FGM media in terms of a decrease in area size after 4 h. Comparatively, ML and MH showed a lower decrease in the area closure among all the media used in this study. After 24 h, complete wound closure was observed (100% confluence) in all the media mentioned.

In different culture systems, cadFibroblasts may have induced the secretion of several different types of growth factors that are involved in the healing of wounds and may have an immediate impact on cell migration and proliferation as several cytokines and growth factors collaborate to control the various phases of tissue restoration during wound healing^20^. However, further study is required to analyze the cellular secretions that may act as a mediator between fibroblasts and the culture conditions.

Several biological systems interact with one another throughout the complicated process of wound healing via cell-to-cell interactions and diffusible substances^21,22^. The timely and ideal operation of a wide range of varied processes that result in the production of new tissue is essential for successful wound healing. Therefore, the fibroblasts are essential for tissue repair and wound healing since they are the major source of the ECM, growth factors, and adhesion molecules essential for tissue growth and angiogenesis^2,28^.

The scratch wound healing study examines the random cell migration as there was no chemotactic gradient. Contrary to this, a chemotactic gradient is used in a transwell migration test to evaluate the directional migration generated between the cell-containing upper chamber suspended in different media and the chemoattractant-containing lower chamber (EGF). With the equal concentration of cells in the upper chamber, a significantly (*p* < 0.05) higher number of cells migrated in DL and DH when compared with ML and MH. Furthermore, cell migration in FGM was significantly (*p* < 0.05) lower when compared with DL, DH, ML, and MH. The slowest migration was observed in FGM media when compared with other media indicates rather than chemotaxis, chemokinesis was involved in the case of FGM. This could be due to the growth factors or supplements present in FGM.

To imitate the action of ECM invasion and extravasation, a sheet of ECM or endothelial cells can be placed on top of the transwell membrane to further analyze how cells can sense a specific chemoattractant and migrate towards it through a physical barrier. Thus, this test can be used to study cell invasion^23^. Additionally, morphological analysis during 3-D invasion after the invasion via Matrigel may benefit from immunological tagging of cytoskeletal proteins in conjunction with fluorescence imaging.

To understand the process of cell migration more precisely, higher temporal and spatial resolution might be used, for example, time-lapse imaging of scratch assays^29^ or by utilizing more sophisticated techniques like traction force microscopy that provide a better understanding of the cell populations at multicellular levels^30^. Fluorescently marked cells can be followed using optical fluorescence microscopy in more complex 3D tissues (for example, by combining optical coherence with multiphoton microscopy)^31^.

The relative fold change in the gene expression was calculated by the ΔΔCT method, as described earlier^32^. The results are presented as the fold-change of target gene (FSP-1 or vimentin) expression in the target samples (cadFibroblasts cultivated in different culture media i.e. DH, ML, MH, and FGM) relative to a reference sample or calibrator (cadFibroblast grown in DL), normalized to a reference gene or endogenous control (GAPDH). The higher expression of the FSP-1 gene was observed in ML compared with the calibrator and other culture media. This suggests the favorable effect of certain component/s of ML that cause specifically much higher expression of the FSP-1 gene in the cadFibroblasts during the culture. However, while comparing the expression level through immunocytochemical analyses, no such higher difference among the groups [ML vs calibrator (DL) and other treatment groups] was observed. The probable reasons for the lack of a strong relationship between mRNA and protein expression level of FSP-1 in the ML group may be due to the difference in the translational regulation of FSP-1 mRNA. This observation necessitates studies to investigate the media components and the marker expression during the process of cell cultivation.

Overall, the results presented in this study convincingly illustrate that high glucose (30 mM) significantly affects the culture characteristics of cadFibroblasts in DMEM/F-12. The findings also indicate the base media-dependent effect of glucose supplementation as it was not observed in MEM.

## Conclusion

In this study, we investigated the effect of different culture media and glucose supplementation on cadFibroblast cells for cell morphology, proliferation, PDT, CFU, wound healing, transwell cell migration, and differential expression of fibroblast-specific markers (FSP-1 and vimentin) through dICC and RT-PCR. The isolated cells had fibroblastic-typical shapes and growth characteristics in all the culture media. The results demonstrate that DMEM/F-12 supports the growth of cadFibroblast in the culture system and can be used for the culture, proliferation, and expansion of cadFibroblast cells. Further, the high glucose supplementation increased the efficiency of DMEM/F-12 and the glucose-induced effect could be base medium dependent as ML and MH did not show any significant difference. Importantly, our study also depicts that *α*-MEM cannot be considered the media of choice for cadFibroblast culture. Based on the differential expression of FSP-1 along with vimentin, FSP-1 can be used as a specific marker for the characterization and enrichment of dermal fibroblasts for future scientific studies and clinical applications of these cells.

## Materials and methods

### Sample collection

For the collection of skin samples, adult Barbari bucks (*Capra hircus*; age ~ 3 years; n = 4) were maintained in the experimental shed of ICAR-Central Institute of Research on Goats, Makhdoom, Mathura, India. All the animals were housed under a semi-intensive management system with ad libitum feed and water. During the period of experimentation, the range of ambient temperature (°F), relative humidity (%), and Temperature Humidity Index (THI) were 83.94–73.63 F, 46.51–54.53, and 76.31–69.72, respectively. In this study, explant material (skin tissue samples; ~ 0.25 cm^2^) was taken from the base of the tail of adult Barbari bucks. After shaving the area beneath the tail, 1.0 ml of 2% Lignocaine hydrochloride was infused near the site of the incision. Further, the area was properly disinfected using a 70% alcohol swab and precisely dissected before transporting the sample aseptically in Dulbecco’s Phosphate Buffered Saline (DPBS) supplemented with antibiotics to the laboratory within 10 min. The Institute Animal Ethics Committee (IAEC) has approved the present investigation. The methods followed in this study were as per the guidelines of Purpose of Control and Supervision on Experiments on Animals (CPCSEA) and in accordance with the ARRIVE guidelines.

### Harvesting of cells

For the isolation of cadFibroblasts, skin samples were soaked once in 70% ethanol and then washed with DPBS (Gibco, Cat#14040-117) containing 10 µg/ml Gentamycin (Sigma-Aldrich, Cat#G1264) and 10 µl/ml antibiotic–antimycotic solution (Sigma-Aldrich, Cat#A5955). Tissue samples were then transferred aseptically to a sterile 90 mm petri dish (Genetix, Cat#10090) containing 500 µl drop of DPBS containing antibiotics using sterile forceps. The epidermal layer of excised skin samples was then scratched with a sterile disposable scalpel and the sample was then again rinsed with 70% ethanol followed by washing with DPBS containing antibiotics. After being extracted, the skin samples were cut into 2–3 mm^2^ pieces with a sterile scalpel and forceps in 500 µl drop of culture media [DMEM/F-12 (Sigma-Aldrich, Cat#D8437)] supplemented with 15% FBS (Gibco, Invitrogen, Carlsbad, CA, USA, Cat#10082-147), 0.5% non-essential amino acid solution (NEAA; Sigma-Aldrich, Cat#M7145), 10 µg/ml Gentamycin (Sigma-Aldrich, Cat#G1264) and 10 µl/ml antibiotic–antimycotic solution (Sigma-Aldrich, Cat#A5955). Small pieces of skin samples were then transferred into the 6-well cell culture plate (Cellstar, Greiner bio-one, Cat#657160) and incubated in a CO_2_ incubator at 38.5 °C, 5% CO_2_ in humidified conditions. The culture plates were left undisturbed for 48 h to avoid the tissue from dislodging. Then, with minimal disturbance, culture plates were checked after 1 wk for cell migration and proliferation from explants using an inverted microscope (Magnus INVI, India).

### Monitoring for contamination and the growth of primary cell cultures

An inverted microscope (10×) was used to examine the cultures every alternate day to evaluate for explant displacement and expansion of primary fibroblast cells around explants. The culture was also examined for any indications of microbial or fungal contamination at higher magnification (40×). On the fifth day, the culture media in the dishes was changed, and on the eighth day, the expansion of cells around the explants was noted. The cells were passaged after reaching 60–70% confluency around the explants.

### Enrichment of cadFibroblasts

The enrichment of cadFibroblasts was performed after primary culture by immunomagnetic separation [magnetic-activated cell sorting (MACS)] method^33^. This was performed using the MiniMACS separation unit (Miltenyi Biotec GmbH, Bergisch Gladbach, Germany; Cat#130-042-102) and MS column (Miltenyi Biotec GmbH, Germany; Cat#130-041-201) following the method described earlier^34^, with minor modifications. Briefly, after partial trypsinization of monolayer after primary culture, cell clumps were removed by passing cell suspension through a pre-separation 60 µm sterile filter. The cell suspension was then washed with 1 ml MACS buffer [PBS (pH 7.4) supplemented with 2 mM EDTA and 0.5% BSA] by centrifugation at 300×*g* for 10 min at 25 °C. After centrifugation, the cell pellet was resuspended in a solution of MACS buffer (80 µl) and rabbit anti-Fibroblast specific Protein-1 polyclonal antibody (10 µl; Merck S100A4; Cat#ABF32) and incubated for 15 min at RT with occasional mixing. Subsequently, the cell suspension was centrifuged (300×*g*, 10 min, 25 °C) and the pellet was dissolved with a solution of MACS buffer (80 µl) and 20 µL of magnetic microbeads labeled secondary antibody [anti-rabbit IgG microbeads (Miltenyi Biotec GmbH, Germany; Cat#130-048-602)] before incubated at 4 °C for 15 min. Then, the cells were again centrifuged (300×*g*, 10 min, 25 °C) and the cell pellet was dissolved in MACS buffer (500 µl). Simultaneously, the MS column was arranged by rinsing with 500 µl of degassed MACS buffer. The cell suspension conjugated with magnetic microbeads was then applied onto the MS column and flow-through was collected in a sterile tube (contained mostly FSP-1-negative cells). Further, the MS column was rinsed three times using 500 µl degassed MS buffer on each occasion. The MS column was then removed from the separator and the FSP-1^+^ cadFibroblasts were flushed out using 1.5 ml MS buffer in a sterile 2 ml microcentrifuge tube. The fraction with magnetically labeled enriched cadFibroblast was used for subsequent culture and trials.

### Establishing secondary cultures

For the secondary culture, cultured cells were carefully rinsed once using DPBS (Gibco, Cat#14190-136). After gentle shaking of the cell culture plate, DPBS was discarded. This was followed by the incubation of a cell sheet (a complete layer of cells) with 1 ml 0.25% Trypsin–EDTA solution (Gibco, Cat#25200-072) for 3–5 min in a CO_2_ incubator at 38.5 °C, 5% CO_2_ in humidified conditions. When seen under a microscope after three min, cadFibroblasts rounded and started to separate from the culture flask's plastic surface. After complete detachment of cells, 2 ml of washing media (DMEM/F-12, supplemented with 10% FBS, 0.5% NEAA, 10 µg/ml Gentamycin and 10 µl/ml antibiotic–antimycotic solution) was dispensed on the cell culture plate, and the cells were gently spread out by pipetting up and down 2–3 times followed by centrifugation at 2655×*g* for 10 min to form a pellet. After that, serial passaging was used to expand these cells. Cells from primary cultures were replicated in T-25 flasks (SPL Lifesciences, Cat#70325) for secondary culture, and further expansion was done in T-75 flasks (SPL Lifesciences, Cat#70375). The cadFibroblast cells at P4–P6 were used for further experiments.

### Experimental design

To detect the effect of culture media, three commercially available culture media viz. DMEM/F-12 (Sigma-Aldrich, Cat#D8437), *α*-MEM (Gibco, Cat#12561-049), and Human Dermal Fibroblast Growth Media (FGM; CELL Applications Inc., Cat#116-500) were obtained and were used with low and high glucose supplementations. CadFibroblast cells were cultured in the five different culture media and grouped as:

1. DL: DMEM/F-12 supplemented with 15% FBS, 0.5% L-Glutamine, 0.5% NEAA, 10 µg/ml Gentamycin, 10 µl/ml antibiotic solution, and low glucose (5.5 mM)
2. DH: DMEM/F-12 supplemented with 15% FBS, 0.5% L-Glutamine, 0.5% NEAA, 10 µg/ml Gentamycin, 10 µl/ml Antibiotic solution, and high glucose (30 mM)
3. ML: *α*-MEM supplemented with 15% FBS, 0.5% L-Glutamine, 0.5% NEAA, 10 µg/ml Gentamycin, 10 µl/ml antibiotic solution, and low glucose (5.5 mM)]
4. MH: *α*-MEM supplemented with 15% FBS, 0.5% L-Glutamine, 0.5% NEAA, 10 µg/ml Gentamycin, 10 µl/ml antibiotic solution, and high glucose (30 mM) and
5. FGM: without supplementation

### Double immunofluorescence (dIF) staining of cadFibroblast cell monolayer

Double immunofluorescence (dIF) staining was carried out as mentioned by Singh et al*.*^34^ with minor alterations. CadFibroblast cells in different culture media (as mentioned above) were seeded in 96-well culture plates (Costar, Corning Incorporated, Cat#3595) and observed until the attainment of 30–50% confluency. At this stage, cells were then fixed with Citrate-Acetone-Formaldehyde fixative solution (100 µl/well) for 30 min at RT followed by the permeabilization of cells with Triton X-100 (0.5%, 100 µl/well) for 30 min. After washing with DPBS (3 washings with 5 min each) blocking solution (2% BSA in PBS, 150 µl/well) was added and incubated for 30 min at RT to prevent non-specific binding. Again, washing with DPBS was given (3 washings with 5 min each) followed by incubation of cells with primary antibodies i.e., anti-vimentin monoclonal antibody [mouse IgG1 (1:200 dilution); Invitrogen RV203; Cat#MAI-06908] and anti-Fibroblast specific Protein-1 [rabbit polyclonal Ab (1:200 dilution); Merck S100A4; Cat#ABF32)] for 1 h at RT. The primary antibody addition in negative controls was omitted. The monolayer was then incubated with secondary antibodies [Alexa Flour 488 donkey anti-mouse (1:200 dilution; Invitrogen, Cat#R37114 and Anti-rabbit IgG Alexa Flour (R) Fab 2 (555); Cell Signalling Technology Cat#4413S] in dark for 30 min at RT. After five washings with DPBS (5 min each) further incubation of monolayer was done with DAPI (1 µg/ml) (Sigma-Aldrich, Cat#D8417) for 1 min in dark at RT before they were observed using a fluorescence microscope (Zeiss Axiovert A1, Germany).

### Measuring cell viability and cell proliferation assay

For these assays, cells were exposed to the standard subculture procedure as mentioned for cell expansion protocol. The equal volume of 0.4% trypan blue solution (1:1) (Gibco, Cat#15250-061) was then combined with the cell suspension and was allowed to stand for 5 min at RT. 10 µl of the cell suspension was then put between the cover slip and the hemocytometer chamber's edge, and the results were assessed immediately using an automatic cell counter (Countess II FL Automated Cell Counter; Thermo Fisher Scientific).

For measuring the effect of culture medium on cell proliferation, after trypsinization (as mentioned above) cells were seeded at P-5 in 2 ml of different culture media in a 6-well culture plate under mentioned culture conditions. Total viable cells in each well were counted each day over 5 days period (by trypsinizing) by automatic cell counter (Countess II FL Automated Cell Counter; ThermoFisher Scientific). The total cell count was calculated to obtain the growth curve and PDT with the use of an online algorithm, PDT during culture for certain time intervals (d 0–3 and d 3–5) (http://doubling-time.com)^35^.

### Colony forming unit (CFU) assay

This assay was performed to investigate whether a single caprine adult fibroblast cell could establish a colony in various growth media. After trypsinization, approximately 50,000 cells per well (P-6) in different culture media were cultivated in 6 well plates with 5% CO_2_ at 38.5 °C in a CO_2_ incubator for 48 h. For CFU assay, seeding of a low number of cells was required to discriminate individual colonies as the counting of CFU assumes that every colony is founded by a single viable cell. After washing with DPBS, colonies were fixed with Citrate-Acetone-Formaldehyde fixative solution for 30 min at RT followed by staining with 1% crystal violet solution (Sigma-Aldrich, Cat#V5265). The colonies were thoroughly washed with distilled water before being examined under an inverted microscope (Zeiss Axiovert A1, Germany). Additionally, a multimode spectrophotometer (Sun Rise, Tecan, Switzerland) fitted with the Magellan data analysis software (v.7.2.0.6) was used to measure absorbance at the wavelength of 590 nm after the stain was solubilized in a 1% SDS solution for 10 min at RT.

### Scratch wound healing assay

This assay was done as per the method described by Jahangiri et al*.*^36^ with minor modifications. Approximately 50,000 cells/well (P-4) in different culture media were cultured in 24 well cell culture plates (SPL Lifesciences, Cat#30024) until nearly 100% confluent to achieve growth arrest. The confluent monolayer was then scraped with a sterile 200 µl pipette tip to produce wounds. This was followed by washing with DPBS to remove detached cells and respective media (DL, DH, ML, MH, and FGM) were again added as per the experimental design. The digital photographs of selected culture areas were taken to track the wound closure at different time points immediately upon scratching (0 h), 2 h, 4 h, and 24 h later. An inverted microscope was used to capture the images (Zeiss Axiovert A1, Germany) with a digital camera (AxioCam ICm 1) and analyzed using an image processing algorithm with the ZEN 3.4 blue software (ZEN lite; version 3.4.91.00000) to measure the area of the scratch. The wound area was measured at different time intervals (as mentioned above) and a decrease in the area with time was calculated.

### Chemoattractant-driven cell invasion/transwell migration assay

Transwell plates (24-well plates with 5.0 µm pore polycarbonate membrane tissue culture and 6.5 mm Insert, Costar, Corning Incorporated, USA, Cat#3421) were utilized in the cell migration assay. Briefly, after trypsinization, cells (1 × 10^6^ cells/well) in different media (100 μl) were placed in the inserts' top chambers. Respective media (600 μl) containing 10 ng/ml epidermal growth factor (EGF) were dispensed to the respective lower chambers of wells as the chemotactic factor. Cells were then incubated in a CO_2_ incubator at 38.5 °C, 5% CO_2_ in humidified conditions, and were allowed to migrate for 48 h. After 48 h, migrating cells on the insert's bottom surface were washed with DPBS, while the cells on the top surface were scraped with a cotton swab. After washing, colonies were then fixed with Citrate-Acetone-Formaldehyde fixative solution for 30 min at RT followed by staining with 1% crystal violet solution (Sigma-Aldrich, Cat#V5265). Migrated cells were then observed at lower magnification (4×) and were counted under a bright field microscope (Nikon Instruments, Inc., Eclipse, TE 2000U, Melville, NY, USA) in ten randomly selected fields of the fixed cells.

### Quantitative differential expression of fibroblast-specific marker genes

The total RNA from cadFibroblasts grown in different culture media was extracted by using RNA extraction reagents (RNAiso Plus; DSS Takara, Cat#9108). After isolation, the pellets of RNA samples were dissolved in 30 µl of RNAase-free water (DSS Takara, Cat#9012) before determination of their purity and quantity using a Bio-photometer (Eppendorf) at ratios 260/280 and 260/230 and evaluation of RNA integrity on an agarose gel (1.4%). The passed samples with purity tests (the pure culture obtained by partial trypsinization and serial passaging) were used for cDNA synthesis using 1.0 µg of RNA using a cDNA synthesis kit (DSS Takara, Cat#6110A) and a thermocycler (C1000™ Thermal Cycler, Biorad). The cDNA samples thus obtained were stored at −70 °C until used for qRT-PCR assay using 2-step shuttle PCR to investigate the profile of differential expression of FSP-1 and vimentin genes. For this, reactions were set up in duplicate using TB Green® Premix Ex Taq™ (DSS Takara, Cat#RR420A) and other components *i.e.,* the respective primers (0.5 pmol final concentration; primer details are presented in Table 3), cDNA template (1.0 µl), and NFW (added to make 20 µl final reaction volume) and analyzed by real-time PCR system (StepOnePlus®; Applied Biosystem, USA). For all the samples, the housekeeping reference gene (GAPDH) and no template control were kept as endogenous control and negative control, respectively. The ΔΔCT method was used to determine absolute quantifications and relative fold-change in the expression of the target genes.

**Table 3:** The primer sequences for qRT-PCR analyses for expression of fibroblast-specific marker genes.

Marker genes	Primer	Primer sequence (5' to 3')	Length	Product size (bp)
FSP-1	Forward	CTGATAAGCAGCCCCGGAAA	20	115
	Reverse	ATCTGGAGGAGCCAGGGTAG	20	
Vimentin	Forward	TGACCTGGAGCGTAAAGTGG	20	250
	Reverse	CCACGCTCTCATACTGCTGA	20	

FSP-1 = Fibroblast Specific Protein-1.

### Statistical analysis

The outcomes were displayed as means ± SEM. Data were evaluated using the Mann–Whitney U test or Student’s t-test to determine statistically significant differences when *p* values < 0.05.

### Ethics declarations

The Institute Animal Ethics Committee (IAEC) has approved the present investigation. The methods followed in this study were as per the guidelines of Purpose of Control and Supervision on Experiments on Animals (CPCSEA) and in accordance with the ARRIVE guidelines.

### Supplementary Information

Supplementary Information.

## Data Availability

The authors declare that the data supporting the findings of this study are available within the article and its supplementary information file.
