# Supplementary material for: Cell culture media dependent in vitro dynamics and culture characteristics of adult caprine dermal fibroblast cells
Source: Sci Rep. 2023 Aug 22;13:13716. doi: 10.1038/s41598-023-38634-4 (PMC10444776; doi:10.1038/s41598-023-38634-4)
Supplement: Supplementary file 1 — Supplementary Information. [file 41598_2023_38634_MOESM1_ESM.docx]

**Supplementary Table 1.** Details of the comparative composition of culture media with high and low glucose level used for cultivation of caprine adult dermal fibroblast cells.

| **Components** | **DL (DMEM/F-12 with low glucose)** | **DH (DMEM/F-12 with high glucose)** | **ML (α-MEM with low glucose)** | **MH (α-MEM with high glucose)** |
| --- | --- | --- | --- | --- |
| FBS | 7.5 ml | 7.5 ml | 7.5 ml | 7.5 ml |
| L-glutamine | 250 µl | 250 µl | 250 µl | 250 µl |
| NEAA | 250 µl | 250 µl | 250 µl | 250 µl |
| Gentamycin | 2.5 mg | 2.5 mg | 2.5 mg | 2.5 mg |
| DMEM/F-12 | 41.5 ml | 41.5 ml | **-** | **-** |
| *α*-MEM | - | - | 41.5 ml | 41.5 ml |
| Antimycotic solution | 500 µl | 500 µl | 500 µl | 500 µl |
| Glucose | 99 mg | 540 mg | 99 mg | 540 mg |
